# Mitochondrial DNA analysis efficiently contributes to the identification of metastatic contralateral breast cancers

**DOI:** 10.1007/s00432-020-03459-5

**Published:** 2020-11-24

**Authors:** Giulia Girolimetti, Lorena Marchio, Antonio De Leo, Miriam Mangiarelli, Laura Benedetta Amato, Simone Zanotti, Mario Taffurelli, Donatella Santini, Giuseppe Gasparre, Claudio Ceccarelli

**Affiliations:** 1 Department of Medical and Surgical Sciences (DIMEC), University of Bologna, 40138 Bologna, Italy; 2 Center for Applied Biomedical Research (CRBA), University of Bologna, 40138 Bologna, Italy; 3 Centro di Studio e Ricerca sulle Neoplasie Ginecologiche, University of Bologna, 40138 Bologna, Italy; 4 Department of Experimental, Diagnostic and Specialty Medicine (DIMES), University of Bologna, 40138 Bologna, Italy; 5 Breast Unit, Department of Woman, Child and Urological Diseases, Sant’Orsola Hospital, University of Bologna, 40138 Bologna, Italy; 6 Operative Unit of Pathology, Sant’Orsola Hospital, 40138 Bologna, Italy; 7 Unit of Medical Genetics, Department of Medical and Surgical Sciences (DIMEC), University Hospital S.Orsola-Malpighi, Via G. Massarenti, 9, 40138 Bologna, BO Italy

**Keywords:** Mitochondrial DNA mutations, Contralateral breast cancers, Breast cancer metastasis

## Abstract

**Purpose:**

In daily practice, a contralateral breast cancer (CBC) is usually considered as a new independent tumor despite the indications of several studies showing that the second neoplasia may be a metastatic spread of the primary tumor. Recognition of clonal masses in the context of multiple synchronous or metachronous tumors is crucial for correct prognosis, therapeutic choice, and patient management. Mitochondrial DNA (mtDNA) sequencing shows high informative potential in the diagnosis of synchronous neoplasms, based on the fact that somatic mtDNA mutations are non-recurrent events, whereas tumors sharing them have a common origin. We here applied this technique to reveal clonality of the CBC with respect to the first tumor.

**Methods:**

We analyzed 30 sample pairs of primary breast cancers and synchronous or metachronous CBCs with detailed clinical information available and compared standard clinico-pathological criteria with mtDNA sequencing to reveal the metastatic nature of CBCs.

**Results:**

MtDNA analysis was informative in 23% of the cases, for which it confirmed a clonal origin of the second tumor. In addition, it allowed to solve two ambiguous cases where histopathological criteria had failed to be conclusive and to suggest a clonal origin for two additional cases that had been classified as independent by pathologists.

**Conclusion:**

Overall, the mtDNA-based classification showed a more accurate predictive power than standard histopathology in identifying cases of metastatic rather than bilateral breast cancers in our cohort, suggesting that mtDNA sequencing may be a more precise and easy-to-use method to be introduced in daily routine to support and improve histopathological diagnoses.

## Introduction

Patients who experienced a breast neoplasia have a higher risk of developing a contralateral breast cancer (CBC) during their lifetime (Chen et al. 1999; Peralta et al. 2000; Goldstein et al. 2003; Raymond and Hogue 2006; Hartman et al. 2007). The risk of CBC after the first diagnosis ranges from 0.7 to 1.8% per year (Peralta et al. 2000) and is two- to sixfold higher than the risk of developing a first primary breast cancer for women in the general population (Chen et al. 1999). This estimate approaches 3% in BRCA1/2 germline mutation carriers (Imyanitov and Kuligina 2020). CBC can be categorized as synchronous or metachronous based on the time window between the first and secondary breast cancer development (Imyanitov and Kuligina 2020). There are no uniform clinical criteria that allow discriminating between bilateral CBC (bilCBC) and metastatic cancer spread to CBC (metCBC). In addition, some of the criteria used have relevant critical issues and can, therefore, not be considered fully reliable (de Dueñas et al. 2014; Schrijver et al. 2018). Furthermore, metCBC is associated to a higher risk of diffuse metastasis and, subsequently, to a poorer survival, whereas a new primary carcinoma is characterized by its own patho-biological features, and a better survival (Haffty et al. 1996; Elkhuizen et al. 1998; Huang et al. 2002). Currently, it is suggested the following pathological features be used to define a second CBC as independent or suspected metastasis of the primary: (1) different/identical histotype/histological grading; (2) presence/absence of an in situ component; and (3) different/identical bioprofiles. When such criteria are inconclusive (4) the presence/absence of distant metastasis; (5) the time interval between primary and second tumor and (6) the stage (UICC/AJCC Stage 3) of the primary must be taken into consideration (Chaudary et al. 1984; Chen et al. 1999; Intra et al. 2004; Komoike et al. 2005). Upon application of these parameters, it is evident that the great majority of cases shares different clinical and histologic patterns between bilateral tumors, suggesting an independent clonal origin (Dawson et al. 1991). In contrast, a plethora of studies in the past two decades pointed to the existence of a common clonal origin for a subgroup of these carcinomas. Different techniques have been used to demonstrate this assertion, starting with genetic approaches such as X-chromosome inactivation (Noguchi et al. 1994; Shibata et al. 1996; Banelli et al. 2010), *TP53* mutations (Janschek et al. 2001), allele imbalance (AI) (Imyanitov et al. 2002; Schlechter et al. 2004), microsatellite size alterations (Saad et al. 2008), loss of heterozygosity (LOH) (Schlechter et al. 2004; Saad et al. 2008), comparative genomic hybridization (CGH) (Teixeira et al. 2004; Brommesson et al. 2008), and DNA methylation profile (Huang et al. 2015). Recently, a clonal relationship between paired CBC and primary breast cancer was determined in a subset of cases using massively parallel sequencing assay (Begg et al. 2018), whole exome and genome sequencing (WES and WGS) (Bao et al. 2015; Klevebring et al. 2015; Alkner et al. 2015) and multi-omics approaches (Biermann et al. 2018). Despite the existence of such an abundant literature, today’s practice is to consider bilateral tumors as two independent entities. In this context, valid, rapid and low-cost approaches for discrimination between a clonal and a new primary lesion are still lacking. Mitochondrial DNA (mtDNA) sequencing was reported as an informative approach to define clonality in simultaneously detected gynecological neoplasia (Guerra et al. 2011, 2014; Girolimetti et al. 2017; Perrone et al. 2018a). Due to its tendency to accumulate non-recurrent mutations in cancer cells (Iommarini et al. 2013; Perrone et al. 2018b), high mtDNA variability has been validated by our group as a tool to identify clonal, metastatic masses. Indeed, mtDNA mutations are considered as common somatic events in cancer tissues (Ju et al. 2014), therefore, the use of a random tumor-specific mtDNA mutation carried by two different cancer lesions of the same patient as a marker of clonality is a robust approach. This is mainly due to the lack of hotspot mutations in mtDNA (Liu et al. 2012; Iommarini et al. 2013), whereby the presence of the same tumor-specific mutation in two primary independent neoplasms is virtually impossible. Here, we apply mtDNA sequencing on a cohort of 30 pairs of breast cancer samples to compare its predictive accuracy against standard histopathological criteria used in the daily practice and retrieve the subset of CBC that ought to be classified as metastasis of the primary breast cancer.

## Materials and methods

### Case series

This study was conducted within the frame of the BIL-BREAST 390/2019/Sper/AOUBo study, approved by the local ethical committee (Comitato Etico di Area Vasta Emilia Centro). Internal review board protocols were followed for collection of samples. The study was performed on 30 patients with synchronous or metachronous bilCBC after collection of informed consent obtained in compliance with the Helsinki Declaration. The mean (± standard deviation) age of patients was 63.9 ± 14.1 years (range from 32 to 88 years), median 63.5 years; all cases were diagnosed at the S. Orsola Hospital, Bologna. An alpha-numeric code (from C1 to C30) was assigned to the cases to maintain anonymity. None of the patients enrolled in this study presented metastatic disease (pM0) at diagnosis. Tumors were considered metachronous when at the time of diagnosis there was an interval of more than 6 months between the first and second tumor. For each tumor, the age of the patient at diagnosis, WHO histological type and grade, presence/absence of an in situ component, pTNM-AJCC stage, immunohistochemical bioprofile (ER, PR, Ki-67 and Her2) were recorded (Table 1). The clinical indication of possible contralateral metastasis was evaluated according to the following parameters: Invasive Lobular Carcinoma (ILC) or Special Type vs Invasive Carcinoma No Special Type (IC NST); presence/absence of an in situ component, Stage 3 at onset in one of the two single contralateral tumors or in the first tumor if metachronous; and bioprofile (RO and Her2 + status). When several indicative and contradictory clinical factors were present, the case was considered clinically non-informative.

**Table 1:** Clinico-pathologic characteristics of primary breast cancers, CBC and tumor clonality

Patient	Age: age at first diagnosis	Primary BC						TI	S/M	CBC						Clinical discordance	Clonality clinical classification	Clonality MtDNA classification
		Histology	pG	pTNM	Stage	In situ	IHC-type			Histology	pG	pTNM	Stage	In situ	IHC-type			
C1	46	IC NST	G2	pT1N0M0	1	Yes	Lum	133	M	ILC	G1	pT1N0M0	1	No	Lum	Histology	bilCBC	N.I
C2	59	IC NST	G3	pT1N1M0	2	No	Lum	14	M	IC NST	G3	pT1N0M1	1	Yes	Lum	In situ	bilCBC	N.I
C3	45	IC NST	G3	pT1N0M0	1	No	Lum-H	69	M	IC NST	G3	pT2N3M0	3	No	TN	IHC	bilCBC	N.I
C4	56	IC NST	G3	pT2N0M0	2	No	Lum	52	M	ILC	G3	pT1N0M0	1	Yes	Lum-H	Histology/IHC/*I-S*	bilCBC	N.I
C5	49	ILC	G2	pT1N0M0	1	Yes	Lum	60	M	IC NST	G2	pT1N0M0	1	No	Lum	Histology	bilCBC	metCBC
C6	54	ILC	G2	pT1N0M0	1	No	Lum	96	M	IC NST	G3	pT2N0M0	2	Yes	Lum	Histology/*I-S*	bilCBC	N.I
C7	57	IC NST	G1	pT1N0M0	1	Yes	Lum	44	M	IC NST	G2	pT1N1M0	2	Yes	Lum	*I-S*	bilCBC	N.I
C8	79	IC NST	G2	pT1N1M0	2	Yes	Lum	48	M	IC NST	G2	pT1N1M0	2	No	Lum	None	N.I	metCBC
C9	81	IC NST	G3	pT1N1M0	2	No	Lum	87	M	IC NST	G1	pT1N0M0	1	Yes	Lum	*I-S*	bilCBC	metCBC
C10	71	IC NST	G1	pT1N0M0	1	No	Lum	72	M	IC NST	G1	pT1N0M0	1	No	Lum	None	N.I	N.I
C11	54	ILC	G1	pT1N0M0	1	No	Lum	0	S	IC NST	G1	pT1N0M0	1	No	Lum	Histology	bilCBC	N.I
C12	57	IC NST	G3	pT3N2M0	3	No	Lum-H	0	S	IC NST	G2	pT2N0M0	2	Yes	Lum	Stage/IHC	Dis/N.I	N.I
C13	64	IC NST	G3	pT1N2M0	3	No	Lum	111	M	IC NST	G2	pT1N0M0	1	No	Lum	Stage	metCBC	metCBC
C14	72	IC NST	G2	pT2N0M0	2	Yes	Lum	0	S	ILC	G3	pT1N2M0	3	No	Lum	Stage/Histology	bilCBC	N.I
C15	48	IC NST	G3	pT3N2M0	3	No	Lum	0	S	IC NST	G3	pT2N1M0	3	No	Lum	Stage	metCBC	N.I
C16	64	IC NST	G3	pT2N1M0	3	No	Lum-H	73	M	IC NST	G1	pT1N0M0	1	Yes	Lum	Stage/IHC/*I-S*	Dis/N.I	N.I
C17	80	IC NST	G2	pT1N1M0	2	Yes	Lum	0	S	IC NST	G3	pT1N1M0	2	Yes	Lum	*I-S*	bilCBC	N.I
C18	73	IC NST	G2	pT1N0M0	1	No	Lum	12	M	IC NST	G2	pT1N0M0	1	No	Her2	IHC	bilCBC	N.I
C19	70	IC NST	G2	pT4N3M0	3	No	Lum	0	S	IC NST	G3	pT1N0M0	1	No	Lum	Stage	metCBC	metCBC
C20	88	IC NST	G1	pT2N0M0	2	No	Lum	14	M	ILC	G1	pT2N0M0	2	Yes	Lum-H	Histology/IHC/*I-S*	bilCBC	N.I
C21	32	IC NST	G3	pT4N2M0	3	No	Lum-H	14	M	IC NST	G3	pT3N2M0	3	No	Lum-H	Stage	metCBC	N.I
C22	82	IC NST	G2	pT1N0M0	1	Yes	Lum	96	M	IC NST	G3	pT2N2M0	3	No	TN	IHC	bilCBC	N.I
C23	88	IC NST	G1	pT2N0M0	2	No	Lum	0	S	IC NST	G1	pT1N0M0	1	No	Lum	None	N.I	N.I
C24	63	ILC	G3	pT1N0M0	1	No	Lum	13	M	IC NST	G3	pT1N0M0	1	No	Lum	Histology	bilCBC	N.I
C25	43	ILC	G3	pT2N0M0	2	No	Lum	90	M	IC NST	G2	pT2N0M0	2	No	Lum	Histology	bilCBC	N.I
C26	72	IC NST	G2	pT2N0M0	2	No	Lum	0	S	IC NST	G2	pT1N0M0	1	Yes	Lum	None	N.I	N.I
C27	78	IC NST	G3	pT2N1M0	3	Yes	TN	0	S	IC NST	G3	pT1N0M0	1	Yes	TN	Stage/*I-S*	Dis/N.I	metCBC
C28	69	IC NST	G2	pT2N0M0	2	No	Lum	0	S	IC NST	G2	pT1N2M0	3	Yes	Lum	Stage	metCBC	N.I
C29	61	IC NST	G3	pT1N1M0	2	No	TN	0	S	IC NST	G3	pT1N2M0	3	Yes	Lum	Stage/IHC	bilCBC	N.I
C30	61	IC NST	G3	pT2N3M0	3	No	Lum	10	M	IC NST	G2	pT1N1M0	2	No	Lum	Stage	metCBC	metCBC

The last two columns on the right report the classification according to clinical parameters and mtDNA analysis

*IC NST* invasive carcinoma no special type, *ILC* invasive lobular carcinoma, *TI* time interval between the two tumors (months), *S/M* synchronous vs. metachronous, *Lum* luminal-like, *Lum-H* luminal-like/Her2 + , *TN* triple negative, *Dis* discordant clinical factors, *N.I*. not informative, *BC* breast cancer, *CBC* contralateral breast cancer, *bilCBC* bilateral CBC, *metCBC* metastatic CBC

### Tumors specimens

All cases were blindly reviewed by two expert pathologists (DS and ADL) with respect to the original diagnosis. For each patient involved in the study, formalin-fixed/paraffin-embedded primary tumors and synchronous or metachronous bilCBC, as well as non-tumor tissue samples were available. To compare the accuracy between a molecular method such as mtDNA sequencing and standard histopathological examination with the aid of immunohistochemical (IHC) analysis, samples from the same paraffin blocks were used. Tissue sections were collected, made anonymous, coded, and sent in a blinded manner for the two parallel investigations. Clinico-pathological classification was based on IHC analysis of the pathologists review and interpretation of morphologic features (on haematoxylin and eosin-stained slides). The two pathologists compiled the classification in bilCBC or metCBC after consensus review of each case. Any discrepancies were resolved by joint viewing at a multi-head microscope.

### MtDNA sequencing

To identify the part of the tissue with a higher percentage of tumor cells, haematoxylin and eosin sections were used. Specific areas of paired tumors and non-tumor tissue were marked on the stained slides. Based on this first analysis, 10 slides of 10-µm sections were prepared from each paraffin tissue block. Five unstained slides were used to harvest the selected area of the tissue using a microscope-guided dissection with a scalpel. Total DNA was extracted with the ReliaPrep™ FFPE gDNA Miniprep System (Promega) according to the manufacturer’s protocols. 10 ng of total DNA was used for mtDNA amplification of 46 contiguous segment using a set of 46 primer pairs as previously described (Girolimetti et al. 2017). KAPA2G Fast PCR Kit (Sigma Aldrich) was used for PCR amplification in a 9700 thermal cycler. The 46 purified PCR products were used for direct sequencing with BigDye kit version 1.1 (Thermo Fisher Scientific) and the sequences were run in an ABI 3730 Genetic Analyzer (Thermo Fisher Scientific). Electropherograms were aligned with rCRS mitochondrial reference sequence using SeqScape version 2.5 software (Applied Biosystems). Detected mtDNA tumor-specific mutations were validated using a second PCR reaction. Furthermore, the same mtDNA variant of interest was confirmed using a second independent extraction of DNA of the same sample from the remaining slides to exclude DNA contamination or sample mix-up. The sequencing of matched unaffected tissues is required to ascertain the tumor-specific origin of mtDNA mutations.

### MtDNA variants analysis

To annotate mitochondrial variants, FASTA files from primary breast cancer and CBC of each patient involved in the study were used as input for MToolBox (Calabrese et al. 2014). The pipeline includes a prioritization of the variants based on the pathogenicity of the mutated allele, the nucleotide variability of each variant site, and amino acid variability (Santorsola et al. 2016). Selected variants where then analyzed using HmtVar (https://www.hmtvar.uniba.it) (Preste et al. 2019). Nucleotide site-specific variability was estimated using HmtVar or MToolBox. Allele frequency (AF) and disease score (DS) were reported from HmtVar (Preste et al. 2019). The pathogenicity of a mutation was established using two different criteria, for non-synonymous variants a DS equal or greater than 0.43 and an AF equal or lower than 0.003264 (DS ≥ 0.43 and AF ≤ 0.003264), for tRNA variants a DS equal or greater than 0.35 and an AF equal or lower than 0.005020 (DS ≥ 0.35 and AF ≤ 0.005020) as previously reported (Preste et al. 2019). Sequences of C1–C30 samples, both primary breast cancers and CBCs, were deposited in the public database (GenBank Accession Numbers MW172442 to MW172501).

## Results

### Clinico-pathological classification of contralateral cases and clonality prediction

Of the 30 patients enrolled in the study, 11 (36.7%) cases were diagnosed with synchronous and 19 (63.3%) cases with metachronous breast cancer. The mean ± S.D. of the time interval between the two metachronous neoplasms was 58.3 ± 38.2 (median 60 months; range 10–133 months). The distribution of the clinico-pathologic parameters considered for the clonal classification of our cases was as follows: histological classification showed 21/30 (70%) cases as bilateral IC NST; in 9/30 (30%) cases, one of the two was an ILC. In synchronous cases, the in situ component was completely absent in 4/11 (36.4%), was present in 1 of paired tumors in 5/11 (45.5%) and in both neoplasias in 2/11 (18.1%) cases, respectively. Seven of 19 (36.8%) metachronous cases presented an in situ component in the second tumor. 7/11 (63.6%) synchronous cases and 4/19 (21.1%) metachronous cases were Stage 3 tumors. For the evaluation of the IHC profile, we considered all ER + /PR + cases as Luminal-like (Lum) without any distinction between subtype A and B. Positive HER2 cases were classified as HER2 if ER-/PR- or Lum-H if ER + and/or PR + . Following these indications, 9/11 (81.8%) synchronous cases had both tumors with the same bioprofile (8 Lum/Lum, 1 TN/TN), and 2/11 (18.2%) had a different bioprofile (1 Lum-H/Lum, 1 TN/Lum). Metachronous cases had 13/19 (68.4%) tumors with the same bioprofile (12 Lum/Lum, 1 Lum-H/Lum-H), while 6/19 (31.6%) were different (3 Lum/Lum-H, 2 TN/Lum-H, and 1 Lum/H) (Table 1).

Overall, therefore, based on clinico-pathologic parameters, the paired neoplasms (bilCBC vs metCBC) were classified as follows: 17/30 (56.7%) cases as independent primary bilCBC tumors; 6/30 (20.0%) cases as metCBC; 7/30 (23.3%) cases as non-informative (N.I.) because lacking specific indications (4 cases), or with informative but contradictory parameters (Dis/N.I.) (3 cases) (Table 1). In particular: 4/11 (36.4%) synchronous and 13/19 (68.4%) metachronous cases were defined as bilCBC; 3/11 (27.2%) synchronous and 3/19 (15.8%) metachronous cases were labeled as clonal metCBC; in 4/11 synchronous (36.4%) and in 3/19 (15.8%) metachronous cases clinico-pathologic parameters were non-informative.

### Mitochondrial DNA sequencing reveals a subset of metCBCs

MtDNA sequencing performed on the 60 samples revealed a total of 36 tumor-specific mutations, i.e. a variant that was not present in the matched non-tumor tissue, in 20/30 (66.6%) cases, with samples harboring up to 3 different ones (Table 2). Nine of such mutations were shown to occur in both paired tumors of 7/30 (23.3%) patients (Table 2), which constituted the set of informative changes that were indicative of a metastatic spread of the primary cancer.

**Table 2:** MtDNA mutations in primary breast cancer, CBC and both tumors

Sample	MtDNA mutations	MtDNA mutation localization	Amino acid substitution	Gene	NV	AF	DS
C1	**m.11529 T > C**	BC	M257T	*MT-ND4*	0.000063 (Preste et al. 2019)	0.0	0.73
C2	m.1415 G > A	BC	–	*MT-RNR1*	0.003988 (Preste et al. 2019)	0.000975	**–**
	m.4107 C > T	BC	–	*MT-ND1*	0.002859 (Preste et al. 2019)	0.000499	–
	m.1646 T > C	CBC	–	*MT-TV*	0.000169 (Preste et al. 2019)	0.000048	0.1
	**m.7937 T > C**	CBC	F118L	*MT-COII*	0.0 (Preste et al. 2019)	0.0	0.8
C3	m.6899 G > A	BC	–	*MT-COI*	0.004346 (Preste et al. 2019)	0.001046	–
C5	m.1982 G > A	BC + CBC	–	*MT-RNR1*	0.0001088	NA	–
C8	m.14207 G > A	BC + CBC	S20N	*MT-ND6*	0.003535 (Preste et al. 2019)	0.000713	0.39
C9	**m.3526 G > A**	CBC	A74T	*MT-ND1*	0.000388 (Preste et al. 2019)	0.000048	0.58
	**m.3833 T > C**	CBC	L176P	*MT-ND1*	0.0 (Preste et al. 2019)	0.0	0.69
	m.16078 A > G	BC + CBC	–	*MT-DLoop*	0.001111 (Preste et al. 2019)	0.000404	–
C10	m.2614 T > C	CBC	–	*MT-RNR2*	0.0001493	NA	–
C12	**m.6642 A > G**	CBC	I247V	*MT-COI*	0.0 (Preste et al. 2019)	0.0	0.67
C13	**m.9591 G > A**	BC + CBC	V129I	*MT-COIII*	0.003358 (Preste et al. 2019)	0.00069	0.55
C14	m.10628 C > T	CBC	–	*MT-ND4L*	0.0 (Preste et al. 2019)	0.0	–
C17	m.3213 A > C	BC	–	*MT-RNR2*	0.001704	NA	–
	m.13151 T > C	CBC	L272P	*MT-ND5*	0.000101 (Preste et al. 2019)	0.000024	0.22
C18	m.2233 T > C	CBC	–	*MT-RNR2*	0,000,096,165	NA	–
	m.3117 C > T	CBC	–	*MT-RNR2*	0,000,015,872	NA	–
C19	m.1743 T > C	CBC	–	*MT-RNR2*	0.0000000063 (Calabrese et al. 2014)	NA	–
	m.3849 G > A	BC + CBC	–	*MT-ND1*	0.014177 (Preste et al. 2019)	0.002996	–
C20	m.2470 G > A	BC	–	*MT-RNR2*	0.000143 (Preste et al. 2019)	0.0	–
	**m.11723 A > T**	BC	T322S	*MT-ND4*	0.0 (Preste et al. 2019)	0.0	0.71
C21	m.15853 C > T	BC	–	*MT-CYB*	0.003306 (Preste et al. 2019)	0.000571	–
	m.1776 G > A	CBC	–	*MT-RNR2*	0.00000862785	NA	–
C22	m.3019 G > A	BC	–	*MT-RNR2*	0.000496 (Preste et al. 2019)	0.000048	–
	**m.9525 G > A**	BC	A107	*MT-COIII*	0.001816 (Preste et al. 2019)	0.000285	0.66
	m.12383 T > C	CBC	I16T	*MT-ND5*	0.0 (Preste et al. 2019)	0.0	0.2
C25	**m.11642 G > A**	CBC	A295T	*MT-ND4*	0.000003 (Preste et al. 2019)	0.000024	0.82
C27	m.5070 A > G	CBC	T201A	*MT-ND2*	0.000095 (Preste et al. 2019)	0.000024	0.22
	m.2492 G > A	BC + CBC	–	*MT-RNR2*	0.0006017671	NA	–
	m.13633 G > A	BC + CBC	G433S	*MT-ND5*	0.000004 (Preste et al. 2019)	0.0	0.29
C28	m.3153 T > C	BC	–	*MT-RNR2*	0.00000000637	NA	–
C30	**m.5212 T > C**	CBC	L248P	*MT-ND2*	0.0 (Preste et al. 2019)	0.0	0.73
	m.1641 G > A	BC + CBC	–	*MT-TV*	0.000074 (Preste et al. 2019)	0.0	0.05
	m.3146 G > A	BC + CBC	–	*MT-RNR2*	0.0003245168	NA	–

All the mitochondrial variants reported in the table are tumor specific. AF, DS and NV were reported from HmtVar (Preste et al. 2019). If mutations were not available in HmtVar, NV was reported from MToolBox analysis. Variants in bold are predicted as pathogenic based on the criteria described in Preste et al. (2019) (for non-synonymous variants: DS ≥ 0.43 and AF ≤ 0.003264; for tRNA variants: DS ≥ 0.35 and AF ≤ 0.005020)

*BC* primary breast cancer, *CBC* contralateral breast cancer, *DS* disease score, *AF* allele frequency, *NV* nucleotide variability

**Fig. 1:**
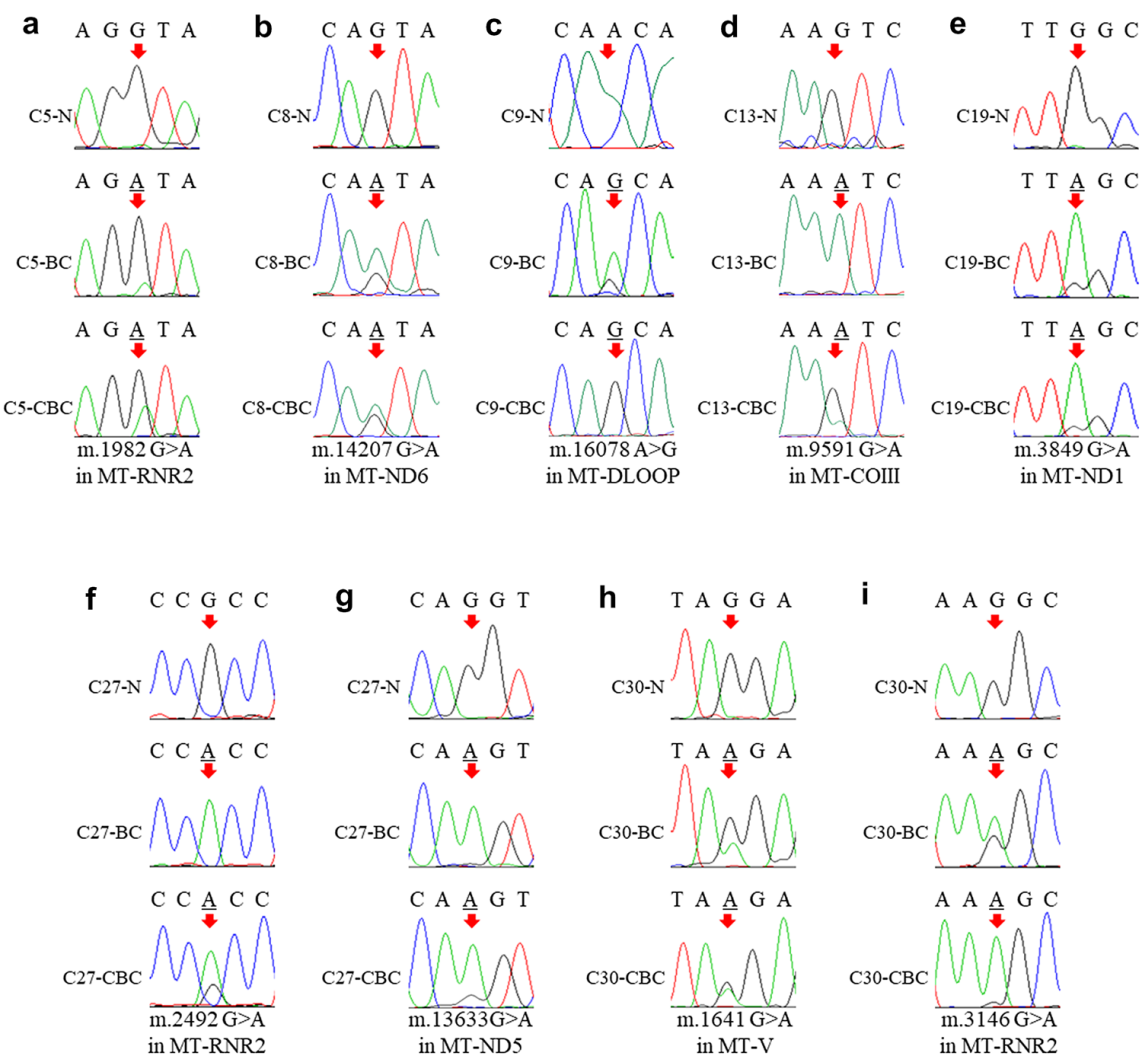
Mitochondrial DNA mutations in primary breast cancer (BC) and CBC tissues. Electropherograms of mitochondrial loci harboring mutations in both BC and CBC (**a**–**i**). Red arrows indicate the mutated bases

We found, in both tumor masses, the m.1982G > A/(*MT-RNR1*) in C5 (Fig. 1a), the m.14207G > A/(*MT-ND6*) in C8 (Fig. 1b), the m.16078A > G/(*MT-DLoop*) in C9 (Fig. 1c), the m.9591G > A/(*MT-COIII*) in C13 (Fig. 1d), the m.3849G > A/(*MT-ND1*) in C19 (Fig. 1e), the m.2492 G > A/(*MT-RNR2*) (Fig. 1f) and the m.13633G > A/(*MT-ND5*) (Fig. 1g) in C27, the m.1641G > A/(*MT-V*) (Fig. 1h) and the m.3146G > A/(*MT-RNR2*) (Fig. 1i) in C30, nearly all of which were detected as heteroplasmic, likely due to an inevitable contamination with non-neoplastic cells of the tumor microenvironment. In the cases that harbored somatic changes in one sample exclusively, we may not rule out that the CBC was a result of a metastatic event, as mtDNA mutations may have occurred after the metastatic spread, or disappeared before, or metastases may have generated starting from non-mutated clones. In such cases, mtDNA was, therefore, not informative.

The informative potential of the two methods for the identification of metCBC resulted to be similar. Indeed, clinico-pathological parameters were informative in 6/30—20% (C13, C15, C19, C21, C28, C30) of cases, while mtDNA analysis in 7/30—23.3% (C5, C8, C9, C13, C19, C27, C30). However, some cases were classified differently by the two methodologies: mtDNA sequencing suggested a clonal origin of CBCs for cases C5 and C9, in contrast with the independent clinico-pathological diagnosis that defined these tumors as bilCBCs. The application of other molecular techniques could be useful to clarify whether mtDNA sequencing or pathological diagnosis was correct, although it is highly unlikely that the same mitochondrial mutation may have occurred independently in primary breast cancer and CBC. In cases C13, C19 and C30, mtDNA analysis was in agreement with clinico-pathological classification, confirming the identification of metCBCs. In cases C8 and C27, where clinico-pathological criteria failed to be conclusive, the presence of the same tumor-specific mtDNA mutations in primary breast cancer and CBC allows to define the masses as metCBC.

## Discussion

Although evidences for tumor clonality should be taken into consideration in the choice of treatment, in daily practice a CBC is considered as a new primary tumor, independent of the first breast cancer and to date no molecular methods are associated with routine iagnostic testing. A substantial literature in the past two decades points to the existence of a percentage of CBCs that are not a second primary but a clonal spread of the first neoplasia. However, patients diagnosed with metastatic breast cancer have a worse prognosis and a different treatment regimen compared to patients with localized disease. The use of pathological parameters is currently the gold standard method to reach a conclusion, but some inherent ambiguities regarding histotype and/or IHC bioprofiles (de Dueñas et al. 2014; Schrijver et al. 2018), tumor heterogeneity or evolution of metastases can complicate the analysis, justifying the implementation of adjuvant molecular techniques in the diagnostic routine.

In the search for clonality, there is no consensus on what is the biological phenomenon to take into consideration, and consequently which type of molecular data need be analyzed to gain the most informative results. Although the literature of the past years lists different molecular techniques, the majority of studies suggested the existence of a subset of cases (from 12 to 39%) in which CBCs resulted to have a metastatic origin. In recent years, WES and WGS have been applied to demonstrate the metastatic nature of recurrent tumors in a wide range of solid cancers, including breast cancer, through the detection of nuclear tumor-specific mutations (Ding et al. 2012, 2013; Haffner et al. 2013; Van Allen et al. 2014). These expensive techniques, particularly cumbersome in their data analysis, yielded indications for clonality in 10–22% of cases, according to whether cohorts of only metachronous, or both synchronous and metachronous tumors were investigated (Klevebring et al. 2015; Alkner et al. 2015; Biermann et al. 2018). Albeit ours was a pilot study, and the cohort relatively limited in size, mtDNA displayed a higher informative potential, reaching 26% within the metachronous-only cohort. Additional advantages of mtDNA sequencing are the need for small amount of DNA and the relative low cost, as well as the standardized protocol, which make this a robust method and a valid approach to infer clonality in clinically ambiguous cases. Furthermore, in genetic studies, the variants to consider to assess clonality need to be non-recurrent mutations in oncogenes to avoid hotspots. Concerning the somatic mtDNA variants, a large fraction reported in the databases such as HmtDB (Clima et al. 2017) and HmtVar (Preste et al. 2019) shows NV and allele frequencies from very low to zero, which implies that two independent masses diagnosed in a single patient may not acquire the same tumor-specific mtDNA mutation by chance. Interestingly, indeed, somatic mutations were shown to be maintained in metachronous tumors even when the time gap between the primary and the second neoplasia was large, such as in cases C5 and C9 (60–86 months, respectively). Such long interval may have caused clinico-pathological parameters to change, whereby the pathologists diagnosis differed from what the mtDNA indicated, suggesting caution must be used in classifying bilCBC based on the time interval between neoplasms.

In our cohort, using mtDNA sequencing, we have found 7/30 patients, diagnosed with two independent CBCs, to be affected by metastatic cancers, accounting for a relevant number of cases that may benefit from a proper diagnosis. In all informative cases, however, we observed the lack of mutations in the primary tumor exclusively, even when multiple changes occurred in the same patient. Conversely, additional mutations to the informative ones, whenever detected, were found in CBCs. This finding suggests that the latter mutations arose subsequently to metastatic spread, on the same mtDNA molecule where the co-occurring somatic variants were mapped. In cases presenting two informative changes, the latter were maintained in the contralateral neoplasia, suggesting they were on the same mtDNA molecule, further reinforcing the clonal origin of the secondary mass, as the probability of the occurrence of two identical somatic mutations is nearly null. Interestingly, 9/30 (30%) cases harbored mutations predicted to be pathogenic (Preste et al. 2019), the majority of which (60%) occurring exclusively in the CBC and only one variant belonging to the informative group of variants, i.e. those present in both paired tumors. Pathogenic mutations were of the missense type, rather than nonsense or frameshift, and 67% of them mapped in Complex I (CI) genes. As missense mutations in CI may cause reactive oxygen species (ROS) overproduction, and ROS were extensively shown to contribute to tumorigenesis (Sabharwal and Schumacker 2014; Moloney and Cotter 2018), this may be suggestive that accumulation of pathogenic mtDNA mutations may be favored in these cancers. Interestingly, only one of the informative mutations was pathogenic, suggesting that their functional relevance in the progression from primary to metastasis may be scarce, and these may well be bystander events that are carried forward during the cancer mass evolution.

It is worth noting that more than 1/3 of the mutations found (13/37) mapped within ribosomal RNA genes, RNR1 and RNR2, prevalently in the latter. For these mutations, a prediction of pathogenicity is not feasible, although their very low variability values are suggestive that they may be functionally relevant. In most cases, rRNA genes mutations contribute to a slow down of the mitochondrially encoded protein synthesis, which may in turn favor a slower replacement of dysfunctional respiratory complex subunits (Porcelli et al. 2016). On one hand, this process may constitute a vicious circle leading to an increase in ROS production, whereas on the other it may promote a pro-tumorigenic Warburg effect by keeping down oxidative phosphorylation. Although this remains to be proven, this may be particularly relevant in those cases harboring CI mutations along with rRNA somatic changes (Table 2).

In conclusion, the application of molecular techniques such as mtDNA analysis allowed the identification of a subset of patients with metastatic CBC. Compared with the use of current standard clinico-pathological classification of CBCs, mtDNA reveals a clonal origin in two cases of CBCs in disagreement with the clinico-pathological diagnosis, and increase the informative potential in cases where histopathological criteria fail to be conclusive. These findings have relevant implications in patient management. The presence of clonal tumors implies a worse prognosis, and distinguishing between a bilCBC and a metCBC is pivotal in determining the most appropriate therapeutic options. As the biology of metastatic cancers is being unraveled, the choice and interpretation of clinico-pathological markers will also require revision and will call for adjuvant molecular techniques easy to implement in diagnostic routines. MtDNA may find its place in such practices, as we previously demonstrated in gynecological malignancies (Guerra et al. 2011, 2014; Girolimetti et al. 2017; Perrone et al. 2018a).

## Data Availability

Mitochondrial DNA sequences were deposited in the public database GenBank (Accession numbers: MW172442 to MW172501).
